# Ternary Copper Tungsten Sulfide (Cu_2_WS_4_) Nanoparticles Obtained through a Solvothermal Approach: A Bi-Functional Electrocatalyst for the Hydrogen Evolution Reaction (HER) and Oxygen Evolution Reaction (OER)

**DOI:** 10.3390/ma16010299

**Published:** 2022-12-28

**Authors:** Mohd. Muddassir, Abdullah Alarifi, Naaser A. Y. Abduh, Waseem Sharaf Saeed, Abdulnasser Mahmoud Karami, Mohd Afzal

**Affiliations:** 1 Department of Chemistry, College of Science, King Saud University, Riyadh 11451, Saudi Arabia; 2 Restorative Dental Sciences Department, College of Dentistry, King Saud University, Riyadh 11545, Saudi Arabia

**Keywords:** OER, HER, electrocatalysis, density of states, Tafel

## Abstract

In this work, Cu_2_WS_4_ nanoparticles have been synthesized via a solvothermal decomposition approach using a heterobimetallic single source precursor, WCu_2_S_4_(PPh_3_)_3_. The single source precursor, WCu_2_S_4_(PPh_3_)_3_, has been characterized using multinuclear NMR spectroscopy, while Cu_2_WS_4_ nanoparticles have been characterized by powder X-ray diffraction (PXRD) for which Rietveld refinement has been performed to authenticate the lattice structure of the decomposed product, Cu_2_WS_4_. Furthermore, FESEM and EDAX analyses have been performed to assess the morphology and composition of Cu_2_WS_4_. An electrochemical study in acidic as well as basic media suggested that Cu_2_WS_4_ nanoparticles possess efficient bifunctional activity towards electrochemical hydrogen as well as oxygen evolution reactions. Linear sweep voltammetry (LSV) performed in 0.5 N H_2_SO_4_ indicates an onset potential for the HER of 462 mV and a Tafel slope of 140 mV dec^−1^. While LSV performed in 0.1 M KOH indicates an onset potential for the OER of 190 mV and a Tafel Slope of 117 mV dec^−1^.

## 1. Introduction

Electrocatalytic water splitting reactions have always been the center of attention amongst researchers as they ensure a green and sustainable approach to mitigating energy shortages, without adverse environmental impacts resulting from the unrestrained use of fossil fuels [1,2,3]. The total water splitting reaction results from the addition of half-cell reactions at the cathode (the hydrogen evolution reaction, or HER) and the anode (the oxygen evolution reaction, or OER). The HER involves a two-electron transfer and is associated with low overpotential and very high efficiency [4,5]. However, the overall rate of water splitting is limited by the OER, owing to its slow kinetics caused by the four-electron transfer involved in O–H bond cleavage and oxygen–oxygen bond formation occurring at a high overpotential [4,6,7,8,9,10,11]. Thus, there is an escalating demand for exploiting state-of-the-art electrocatalysts in order to alleviate the overpotentials associated with HERs as well as OERs [12]. Currently, Pt/C electrocatalysts are reported to be the best HER electrocatalyst [13,14], and RuO_2_ and IrO_2_ represent the most efficient electrocatalysts for OERs [6,7,15]. However, the commercial viability of these precious metal-based electrocatalysts is restricted due to their high price and scarcity. Therefore, significant efforts have been devoted to developing cost-effective transition metal-based catalysts viz. dichalogenide [16,17], phosphide [18,19], nitride [20] and carbide [21] which display superior HER activity. Phosphides [22,23,24], sulfides [25] and oxides/hydroxides [26,27,28,29] have exhibited exceptional OER activities. Commonly, most of the noble metal-free HER catalysts show activity in acidic media [30,31], while OER catalysts operate in alkaline media [2,32]. Thus, it is desirable to design a bifunctional electrocatalyst capable of catalyzing both HERs and OERs in both media to reduce the manufacturing cost of novel water splitting electrocatalysts and decrease the possibility of cross-contamination during long cycles [33]. In this context, multicomponent transition metal-based catalysts are thought to be the perfect candidate because of their variable and high redox states and better conducting properties compared to their single component counterparts [34,35]. For this reason, utilization of ternary transition metal chalcogenides (LTMCs) with layered frameworks as bifunctional electrocatalysts for overall splitting of water has emerged as a promising solution. However, the studies on this class of materials are relatively few, due to the difficulties encountered in their syntheses and thus in obtain high-quality materials [36].

In view of this, layered ternary copper tungsten sulfide (Cu_2_WS_4_), the first member of the LTMC class [37], possesses high electrical conductivity and a wide band gap, thereby making it suitable for electrocatalytic as well as photocatalytic hydrogen evolution reactions, visible light-assisted photocatalysis, electrochemical energy storage devices and biological applications [38,39,40,41,42,43,44,45,46,47,48,49]. Wu and co-workers have reported that single-layer Cu_2_WS_4_ exhibits efficient two-dimensional electrocatalytic activity towards HER [38]. Jing et al. have reported the photocatalytic HER activity of decahedral copper tungsten sulfide [39]. Ozel and colleagues demonstrated that Cu_2_WS_4_ nanocubes can efficiently catalyze a HER with the organic reducing agent decamethylferrocene (DMFc) in a water/1,2-dichloroethane (DCE) biphasic system [40]. For photocatalytic HER, Aslan et al. employed a system consisting of a Cu_2_WS_4_ catalyst and donor; the bridge-acceptor organic dye-sensitized TiO_2_ [41]. Additionally, Patir’s group have reported the use of Cu_2_WS_4_ as a co-catalyst for donor-π-acceptor dye-sensitized photoelectrochemical and photocatalytic HERs [42].

Despite several reports on the HER activity of Cu_2_WS_4_-based materials, there are few studies demonstrating their potential in OERs. For instance, Novak et al. reported that although pristine Cu_2_WS_4_ displays almost negligible activity towards OER, P-doped Cu_2_WS_4_ showed significantly improved OER performance [43]. In addition, recently, Sharma and co-workers have explored the use of porous Cu_2_WS_4_ as a negative electrode in supercapacitors [44]. Pazhamalai et al. have reported Cu_2_WS_4_ anchored on Ni foam to be a highly active negative asymmetrical supercapacitor electrode [45]. Furthermore, Gulen and co-workers employed Cu_2_WS_4_ nanocube inks as the catalyst at the counter electrode (CE) to act as a substitute for more expensive Pt-based CEs, to fabricate a cheap/high efficiency dye-sensitized solar cell (DSSC) [46]. In addition, Jia et al. reported that Cu_2_WS_4_ crystallites exhibited high efficiency towards the visible-light driven photocatalytic reduction of aqueous Cr(VI) [47]. Recently, Kannan et al. reported the excellent antibacterial activity of Cu_2_WS_4_ against harmful bacteria that cause skin cancer [48]. Furthermore, Shan et al. also demonstrated that Cu_2_WS_4_ nanocrystals can be potential antibacterial agents owing to their excellent antibacterial activity and biocompatibility [49]. New, cost-effective electrode materials for accelerating OERs are a target of research; therefore, the application of Cu_2_MoS_4_ and its composites as electrode materials to catalyze the OER [50], and the use of the Ni dithiolate anion and its composites with 2D materials as electrode materials for accelerating OER have both been reported [51].

Previous reports have indicated that Cu_2_WS_4_ and its derivatives/composites possess exceptional catalytic activity towards HERs, but its application towards OERs have been sporadically reported. This inspired us to explore its potential as a bi-functional catalyst to drive HERs in acidic media and OERs in alkaline media. In view of these noteworthy aspects associated with Cu_2_WS_4_, herein we describe the synthesis of Cu_2_WS_4_ (**CWS**), obtained via solvothermal decomposition of a heterobimetallic single source precursor, WCu_2_S_4_(PPh_3_)_3_ (**SSP**) [52]. Additionally, the characteristics of **CWS** as an electrocatalyst for HERs and OERs are described. To the best of our knowledge, this is the first report on the synthesis of Cu_2_WS_4_ using a single source precursor approach and the application of this material as a bifunctional catalyst for OERs and HERs. The pertinent outcomes of this entire investigation are presented herewith.

## 2. Results and Discussion

### 2.1. Synthesis of WCu_2_S_4_(PPh_3_)_3_ (SSP)

**Scheme 1:**
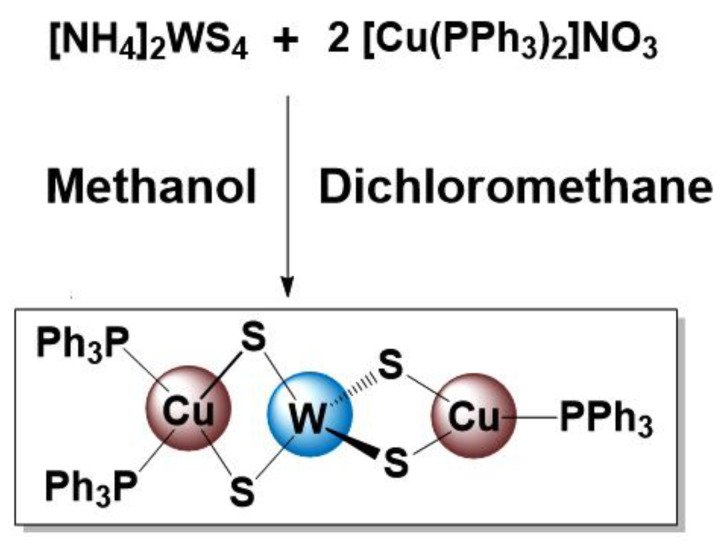
Synthetic routes for the preparation of **SSP**.

The precursor, WCu_2_S_4_(PPh_3_)_3_ (**SSP**) [50], was prepared by development of a new synthetic pathway by reacting diammonium tetrathiotungstate, (NH_4_)_2_WS_4_, with copper triphenylphosphine nitrate, Cu(PPh_3_)_2_NO_3_, in a stoichiometric ratio of 1:2 in a methanol–dichloromethane mixture (Scheme 1). The obtained **SSP** was air-stable and moisture-stable, and its purity and composition were assessed with the help of NMR spectroscopy.

### 2.2. Spectroscopy

The ^1^H NMR spectrum of **SSP** displayed a broad multiplet ranging between δ 7.27–7.47 arising due to the aromatic ring protons of the triphenylphosphine ligands. In addition, the aromatic carbons of the triphenylphosphine ligands displayed signals in the range of δ 128.7–134.2 in the ^13^C NMR spectrum of **SSP**. The ^31^P{^1^H} NMR spectrum displays only one signal at δ 8.67.

### 2.3. PXRD and SEM

**Figure 1:**
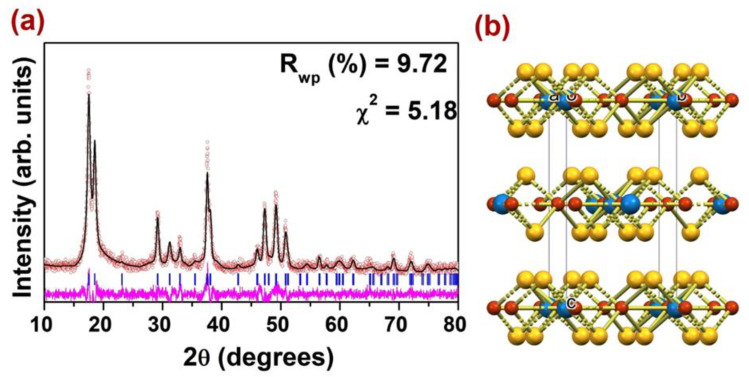
(**a**) The PXRD plot for Cu_2_WS_4_; (**b**) Unit cell view of Cu_2_WS_4_.

The phase formation and structure analysis of Cu_2_WS_4_ were carried out using powder X-ray diffraction (PXRD) data. The data were collected in 2θ range between 10 and 80° with a step size 0.02. The XRD pattern reveals formation of single-phase Cu_2_WS_4_ and the systematic absences in the diffraction pattern are consistent with the body-centered phase of Cu_2_WS_4_. For a detailed structural analysis, the Rietveld refinement of PXRD data was carried out using the tetragonal symmetry in the *I*$\bar{4}$*2 m* space group. Results of the Rietveld refinement of PXRD data of Cu_2_WS_4_ are shown in Figure 1. The fit between the observed and calculated diffraction profile is good, with an almost flat difference profile in the given 2θ range. The lattice parameters of Cu_2_WS_4_ obtained after the Rietveld analysis of PXRD were a = 5.4372(6) Å and c = 10.120(2) Å.

**Figure 2:**
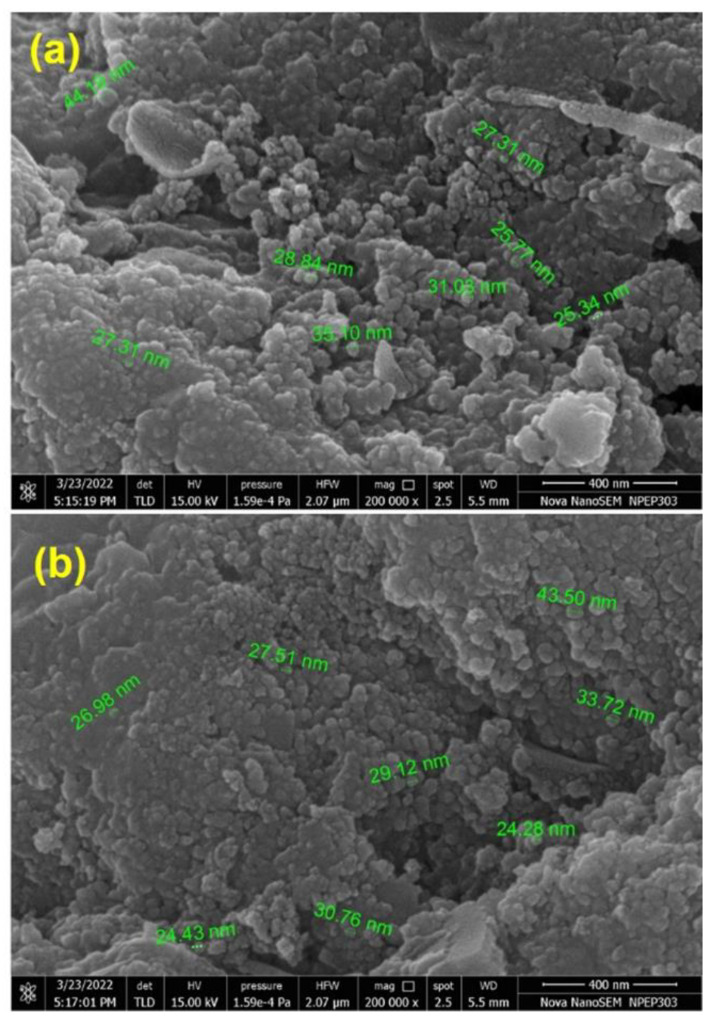
FESEM images for Cu_2_WS_4_ in different region.

Furthermore, SEM analysis revealed that the obtained Cu_2_WS_4_ possess nearly evenly distributed globule-like morphology with individual dimensions of globules ranging between ca. 25 and 30 nm (Figure 2). Additionally, EDAX analysis revealed that the composition of the materials was in agreement with the composition of Cu_2_WS_4_ (Figure S8).

### 2.4. Electrochemical Study

To study the electrocatalytic behaviour of Cu_2_WS_4_ towards electrochemical water splitting, electrochemical measurements were performed in various media such as 0.1 M H_2_SO_4_, 0.5 M H_2_SO_4_ and 0.1 M KOH solution.

**Figure 3:**
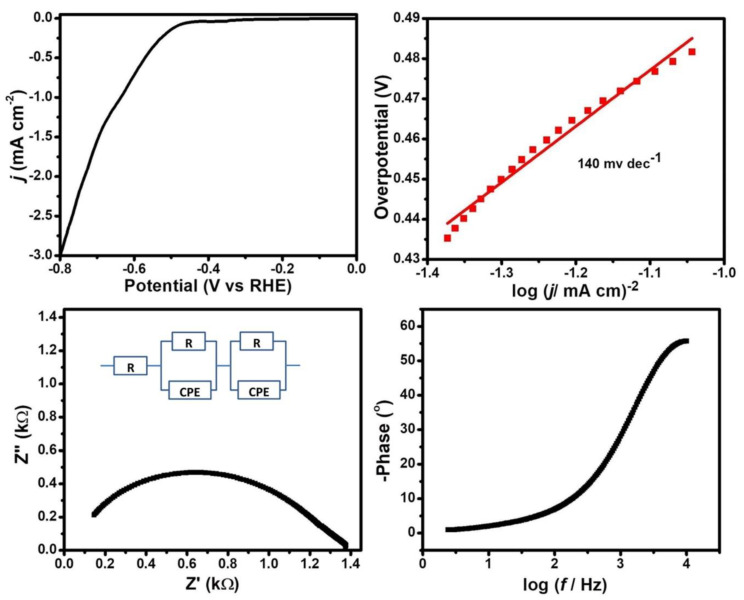
(**Top**) LSV plot at 1 mv sec^−1^ scan rate and corresponding Tafel plot and (**bottom**) Nyquist (with equivalent circuit diagram) and Bode plots from the EIS study of Cu_2_WS_4_ in 0.5 M H_2_SO_4_ solution.

The LSV profile of Cu_2_WS_4_ in acidic media suggested that it exhibits moderate activity towards HER with an onset potential of 462 mV and a Tafel slope of 140 mV dec^−1^ (Figure 3).

H_3_O^+^ + e^−^ + M → M-H_ads_ + H_2_O (1)

M-H_ads_ + M-H_ads_ → H_2_ (2)

M-H_ads_ + H + e^−^ → H_2_ (3)

The Tafel slope is an important criterion to predict the HER mechanism which may proceed via the Volmer–Heyrovsky or the Tafel mechanism based on two possible desorption steps involved in acidic aqueous medium. Usually, a fast discharge reaction (Equation (1)) followed by a rate limiting combination reaction (Equation (2)) leads to a Tafel slope of ~30 mV dec^−1^. When Step 1 is fast and followed by a slow electrochemical desorption reaction, Step 3, a Tafel slope of ~40 mV dec^−1^ is obtained. If Equation (1) is rate-limiting or the surface coverage is close to one, the Tafel slope is ~120 mV dec^−1^. A high Tafel slope (>~120 mV dec^−1^) value indicates a Tafel mechanism for HER.

The LSV study at different concentrations of H_2_SO_4_ suggests there is no change in onset potential (potential where tangents corresponding to faradaic and non-faradaic zones intersect). However, the current density increases at higher H_2_SO_4_ concentrations, which is consistent with the first step as the rate limiting condition (Figure 3, Figures S1 and S2). The LSV profiles at different scan rates for both concentrations of H_2_SO_4_ indicate that current density differs with scan rate, but is not proportional to change in scan rate. Furthermore, the literature reports suggest that the HER mechanism is controlled by the Volmer, Heyrovsky or Tafel mechanisms if the Tafel slope is close to 120, 40 or 30 mV dec^−1^, respectively [53,54,55,56]. Hence, the Tafel slope value of 140 mV dec^−1^ indicates that the Tafel step is rate-controlling for HER reactions catalyzed by Cu_2_WS_4_. Furthermore, the impedance study suggests that the charge transfer resistance (R_ct_) is also dependent on the concentration of H_2_SO_4_ and is of the order of kiloohms (1.2 kΩ in 0.5 M H_2_SO_4_ and 7.1 in 0.1 M H_2_SO_4_ (Figure 3, Figures S3 and S4).

**Figure 4:**
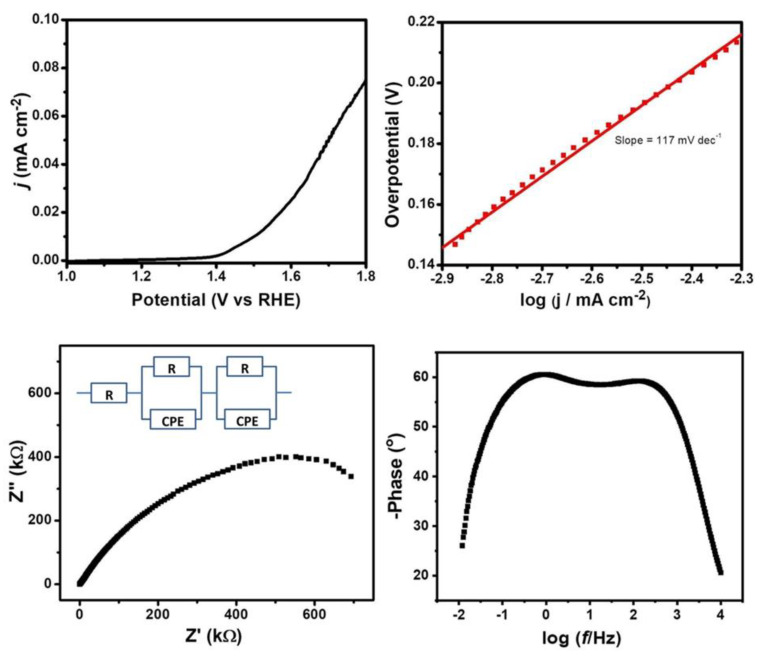
(**Top**) LSV plot at 10 mv sec^−1^ scan rate and corresponding Tafel plot and (**bottom**) Nyquist (with equivalent circuit diagram) and Bode plots from the EIS study of Cu_2_WS_4_ in 0.1 M KOH solution.

In addition to determining the possibility of the application of Cu_2_WS_4_ as an electrocatalyst for HERs, electrochemical measurements were also performed in 0.1 M KOH to check the activity of Cu_2_WS_4_ towards the electrochemical oxygen evolution reaction. The LSV profile of Cu_2_WS_4_ in alkaline medium suggested that the material exhibited activity towards the oxygen evolution reaction with an onset potential 190 mV and a Tafel slope of 117 mV dec^−1^ (Figure 4). Additionally, the impedance study suggests that the charge transfer resistance, R_ct_, is 700 kΩ. The magnitudes of overpotential, the Tafel slope and the Rct suggested that Butler–Volmer kinetics apply to this reaction. As reported earlier, the OER is expected to proceed on the catalyst surface by electrochemically produced metal cations as the active sites via the following steps:

M + OH^−^ → M * − OH + e^−^ (4)

M * − OH + OH^−^ → M * − O + H_2_O + e^−^ (5)

M * − O + OH^−^ → M * − OOH + e^−^ (6)

M * − OOH + OH^−^ → M * − OO + H_2_O + e^−^ (7)

M * − OO → M + O_2_ (8)

Overall reaction: 4OH^−^ → O_2_ + 2H_2_O + 4e^−^ (9)

LSV experiments performed at different scan rates in 0.1 M KOH indicated that the current density varies with scan rate; however, it is not proportional to the change in scan rate (Figure S5). A lower onset and lower Tafel slope for the OER reaction compared to the HER reaction suggest that the catalyst is prone to catalyze the OER reaction at lower onset potential compared to the HER. On the contrary, if we look at the current density and charge transfer resistance, the current density is lower and R_ct_ is higher in the case of the OER compared to the HER. Hence, it can be concluded that although the onset potential for the the OER is lower compared to the HER, the turnover number for the OER is lower than the HER for a given overpotential above the onset potential.

Overall, the most important aspect of employing Cu_2_WS_4_ as a potential bifunctional catalyst for the electrochemical HER reaction in acidic media and the OER reaction in basic media is its very low onset potential. Hence, this material may be a good catalyst for the application of water as potential source of renewable energy in the form of hydrogen.

Furthermore, the stability of the prepared catalyst Cu_2_WS_4_ has been determined by a chronoamperometric study at potentiostatic conditions at different potentials. As shown in Figure S6 (Supplementary Materials), Cu_2_WS_4_ displays good stability with sustainable activity for 30 min at varied potentials. Hence, the catalyst may be used at various potentials for water splitting without change in activity.

Interestingly, in all the EIS spectra of Cu_2_WS_4_ in different media, i.e., 0.1 M H_2_SO_4_, 0.5 M H_2_SO_4_ and 0.1 M KOH, fitted well with R(QR)(QR) equivalent circuit (Figure 3 and Figure 4) (Where Q = constant phase element), with related fitting parameters shown in Table 1. The resistance of the solution decreases with increase in acid concentration due to the high mobility of H^+^ ions. R_c_, Q_c_, R_ct_ and Q_ct_ are resistance and capacitance due to physical barrier properties of the modified electrode (Layer) and charge transfer resistance and capacitance, respectively [57].

The OER performance of the electrocatalyst Cu_2_WS_4_ has been compared with the OER performance exhibited by previously reported electrocatalysts (Table 2). The results indicate that the reported synthesized electrocatalyst offers better performance than the previously reported electrocatalysts [43]. Additionally, the pristine Cu_2_WS_4_ material synthesized previously was found to be almost inactive in OERs. This suggests that the obtained material can be an apt electrocatalytic material for water splitting reactions.

## 3. Conclusions

From this investigation it can be concluded that Cu_2_WS_4_ nanoparticles can be easily synthesized via a solvothermal approach using a heterobimetallic single source precursor, WCu_2_S_4_(PPh_3_)_3_. Electrochemical studies suggested that the prepared nanoparticle was appropriate for bifunctional activation of water to produce hydrogen and oxygen under suitable conditions. The electrochemical activity also suggested that the prepared catalyst possesses better activity towards OERs compared to HERs in terms of onset potential and the Tafel slope. However, it showed poor activity for OERs compared to HERs in terms of current density and charge transfer resistance. Such an investigation will create interest in creating similar strategies for the development of bifunctional catalysts with tungsten, copper and sulfur centers, that might be used to create cutting-edge bifunctional electrocatalysts effective concomitantly in HERs and OERs.

## 4. Experimental

### 4.1. Materials and Methods

All reagents and compounds did not require any additional purification beyond what was performed before use. Both (NH_4_)_2_WS_4_ [58] and Cu(PPh_3_)_2_NO_3_ [59] were synthesized using methods described in the literature. The ^1^H, ^13^C, and ^31^P NMR data were collected using a Bruker Avance IIIHD spectrometer. TMS was used as an internal standard for ^1^H and ^13^C NMR, whereas phosphoric acid was used for ^31^P NMR, and chemical shifts were reported in parts per million (ppm). Pananalytical X-ray diffraction (PXRD) phase identification of the decomposed material used Ni-filtered Cu Kα1 radiation (λ = 1.5405 Å). Glassy carbon electrodes (3 mm dia), Pt wire counter electrodes and Ag/AgCl reference electrodes were used in the electrochemical measurements. All of the electrochemical measurements were conducted on an Autolab 2.0 electrochemical workstation.

### 4.2. Syntheses

#### 4.2.1. Synthesis of SSP (WCu_2_S_4_(PPh_3_)_3_)

A dichloromethane solution of Cu(PPh_3_)_2_NO_3_ (5.200 g, 8 mmol) was added dropwise to a methanolic solution of (NH_4_)_2_WS_4_ (1.392 g, 4 mmol) to yield a yellow precipitate. A yellow solution was obtained after the reaction mixture was stirred for 2 h in a nitrogen environment and then pump-dried. The triphenylphosphine impurities that were released during complex formation were removed by adding about 60 mL of diethyl ether to the yellow residue and stirring it for about an hour. Thereafter, the precipitate was filtered and vacuum-dried.

Characterization data: Yellow powder (yield 4.305 g, 87.80%); M. P. 238 °C, ^1^H NMR (CDCl_3_, 300 MHz, δ): *δ* 7.47, 7.35, 7.27 (m, C_6_H_5_). ^13^C NMR (75 MHz, CDCl_3_) *δ*: 134.2, 133.9 (*p-*C, C_6_H_5_), 130.1 (*o-*C, C_6_H_5_), 128.8, 128.7 (*P-*C, C_6_H_5_). ^31^P NMR (121.54 MHz), *δ*: 8.67.

#### 4.2.2. Synthesis of Cu_2_WS_4_ Nanoparticles

Into a three-necked round-bottom flask attached to a condenser, 500 mg of **SSP** (WCu_2_S_4_(PPh_3_)_3_) was added to ~10 g hot octadecyl amine (ODA) solution at 350 °C under nitrogen, and an orange colored suspension was obtained after 60 min. After 120 min, the reaction was quenched and allowed to cool to room temperature by standing overnight under nitrogen. Excess of chloroform was added to the mixture to flocculate the Cu_2_WS_4_ nanoparticles. The ODA-capped copper tungsten sulfide nanoparticles were separated by centrifugation in chloroform four times and the material was further purified by washing several times with acetone.

### 4.3. Electrochemical Studies of Cu_2_WS_4_ Nanoparticles

Catalytically modified glassy carbon (GC) electrodes were used to take electrochemical readings in 0.1 M H_2_SO_4_, 0.5 M H_2_SO_4_ and 0.1 M KOH solutions. Typically, 5 mg of the decomposition product was dispersed in a 1 mL 4:1 *v*/*v* ethanol–water mixture, and then blended with 20 μL 5 wt.% Nafion. The mixture was sonicated for 10 min, thereafter, 10 μL of the resulting homogenous ink was drop-cast onto the surface of the GC electrode and left to dry at room temperature. The working electrode was catalytically modified GC, the counter electrode was Pt wire and the reference electrode was Ag/AgCl (saturated with KCl). The OER capabilities of the produced nanocatalysts were measured using a linear sweep voltammetry (LSV) approach with scan rates of 2, 5, 10 and 20 mV s^−1^. All reported potentials were calibrated against the reversible hydrogen electrode (RHE). The potential was converted from Ag/AgCl to RHE using Equation (10).

E_RHE_ = E_Ag/AgCl_ + 0.059pH + E_Ag/AgCl_^0^ (10)

(E_Ag/AgCl_^0^ = +0.197V)

Using Equation (2), we determined the overpotential (*η*) for a variety of current densities (*x*).

*η^x^* = E_RHE_ − 1.23 (11)

## Figures and Tables

**Table 1:** Parameters obtained by fitting EIS data with R(QR)(QR) equivalent circuit.

Solution	R_s_ (Ω)	Q_c_		R_c_ (Ω)	Q_ct_		R_ct_ (Ω)
		Y_o_ (10^−6^)	n		Y (10^−7^)	n	
0.5 M H_2_SO_4_	46.1	4711	0.8	327.7	2.826	0.8	1024
0.1 M H_2_SO_4_	967.5	0.3271	0.8436	3369	65.29	0.6714	3844
0.1 M KOH	138.1	2.455	0.7097	1215 × 10^3^	29.85	0.7679	1622 × 10^2^

**Table 2:** Electrocatalytic OER performance of Cu_2_WS_4_ reported herein and previously reported analogous materials.

Material	Onset Potential (V)	Tafel Slope (mV dec^−1^)	Ref.
Cu_2_WS_4_	--	--	[43]
Cu_2_WS_3.4_P_0.6_	1.59	194	[43]
Cu_2_WS_3.42_P_0.8_	1.67	341	[43]
Cu_2_WS_3.6_P_0.4_	1.70	272	[43]
Cu_2_WS_4_	1.42	117	This work

## Supplementary Materials

The following supporting information can be downloaded at: https://www.mdpi.com/article/10.3390/ma16010299/s1, The Supporting Information contains figures related to the electrochemical study and band structure of Cu_2_WS_4_, as well as discussion related to DFT calculations with references. Additionally, it contains the crystallographic information file (cif) of Cu_2_WS_4_ obtained after Rietveld refinement. References [60,61] are cited in the Supplementary Materials.
