# Supplementary material for: Ternary Copper Tungsten Sulfide (Cu2WS4) Nanoparticles Obtained through a Solvothermal Approach: A Bi-Functional Electrocatalyst for the Hydrogen Evolution Reaction (HER) and Oxygen Evolution Reaction (OER)
Source: Materials (Basel). 2022 Dec 28;16(1):299. doi: 10.3390/ma16010299 (PMC9822453; doi:10.3390/ma16010299)
Supplement: Supplementary file 1 [file materials-16-00299-s001.zip › materials-2074379-supplementary.pdf]

Supporting Information

Ternary Copper Tungsten Sulfide (Cu_2WS_4) Nanoparticles Obtained through a Solvothermal Approach: A Bi-Functional Electrocatalyst for the Hydrogen Evolution Reaction (HER) and Oxygen Evolution Reaction (OER)

Mohd. Muddassir [1,*], Abdullah Alarifi [1], Naaser A. Y. Abduh [1], Waseem Sharaf Saeed [2], Abdulnasser Mahmoud Karami [1] and Mohd Afzal [1]

[1] Department of Chemistry, College of Science, King Saud University, Riyadh 11451, Saudi Arabia
[2] Restorative Dental Sciences Department, College of Dentistry, King Saud University, Riyadh 11545, Saudi Arabia
* Correspondence: muddassir@ksu.edu.sa

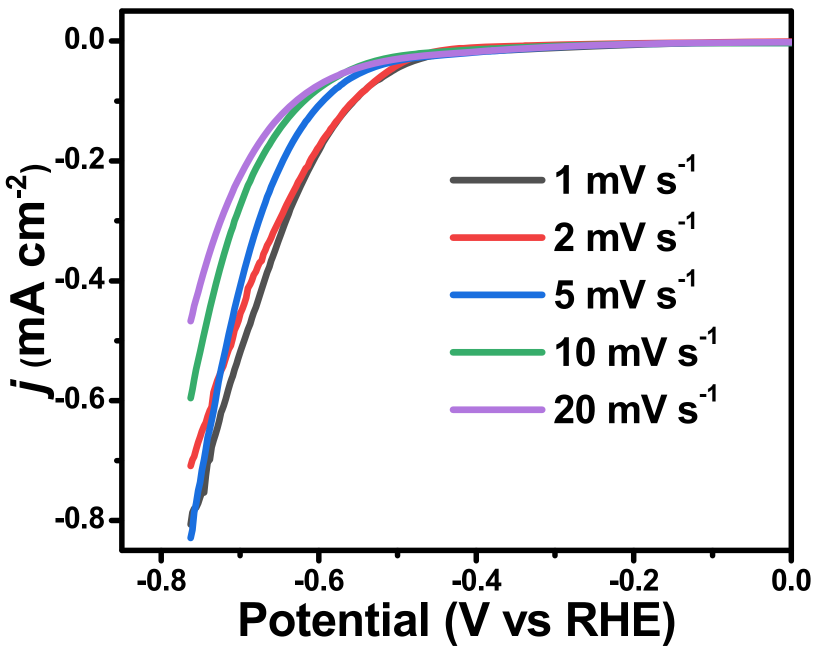

Figure S1. LSV profile of Cu_2WS_4 at various scan rates in 0.1 N H_2SO_4.

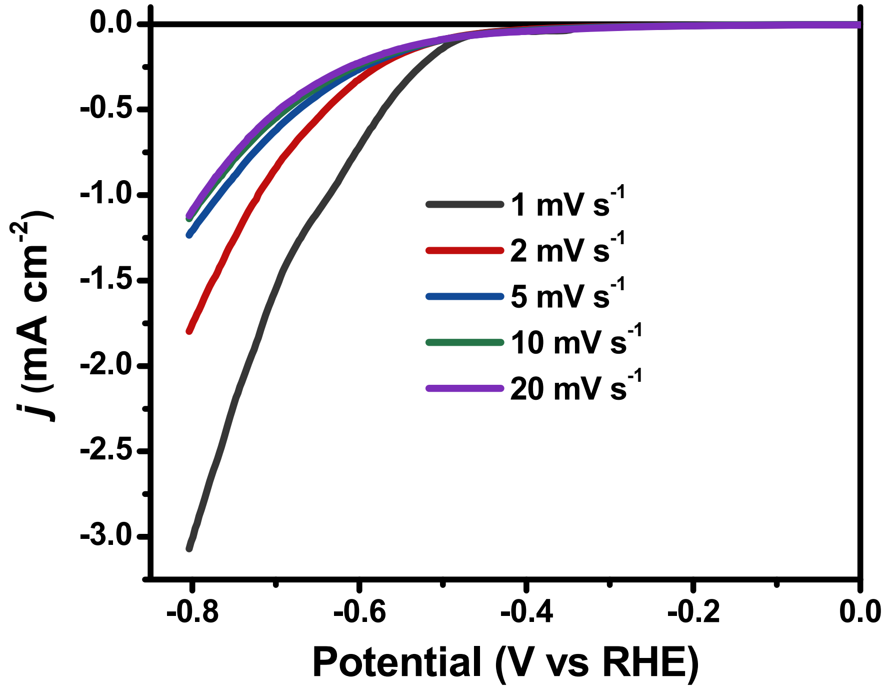

Figure S2. LSV profile of Cu_2WS_4 at various scan rates in 0.5 N H_2SO_4

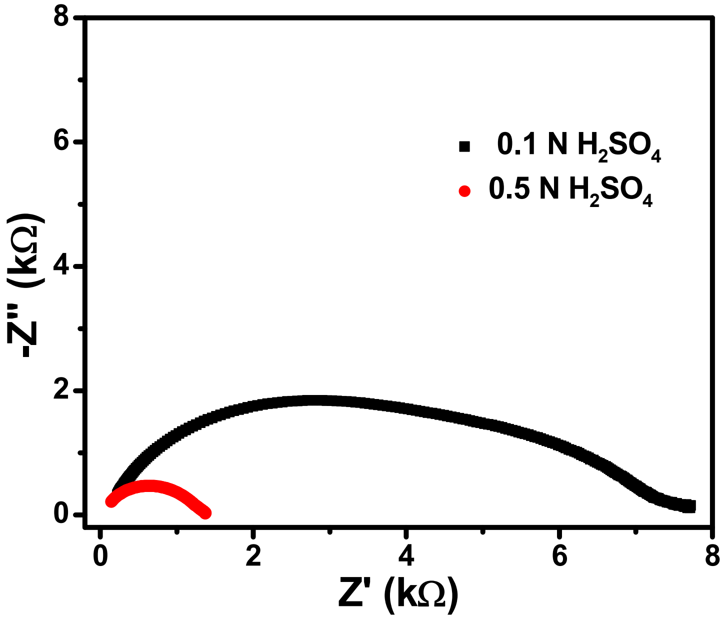

Figure S3. Nyquist plot of Cu_2WS_4 at different acid concentrations.

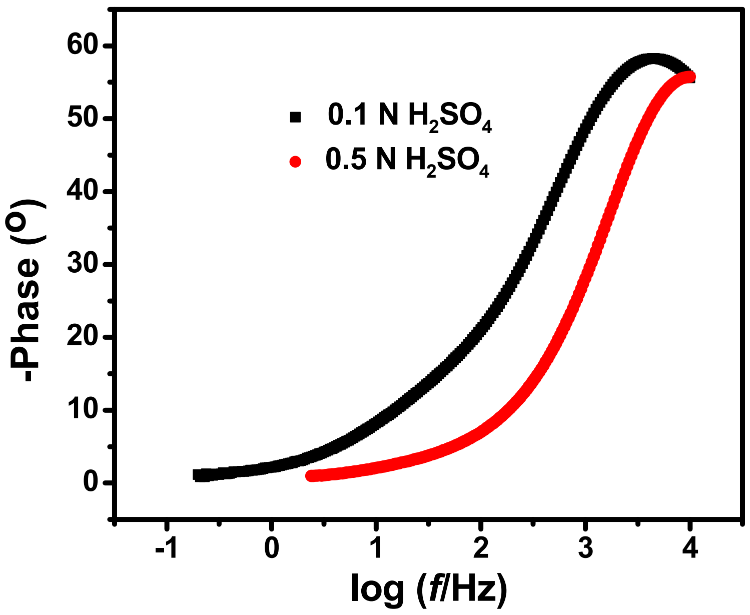

Figure S4. Bode plot of Cu_2WS_4 at different acid concentrations.

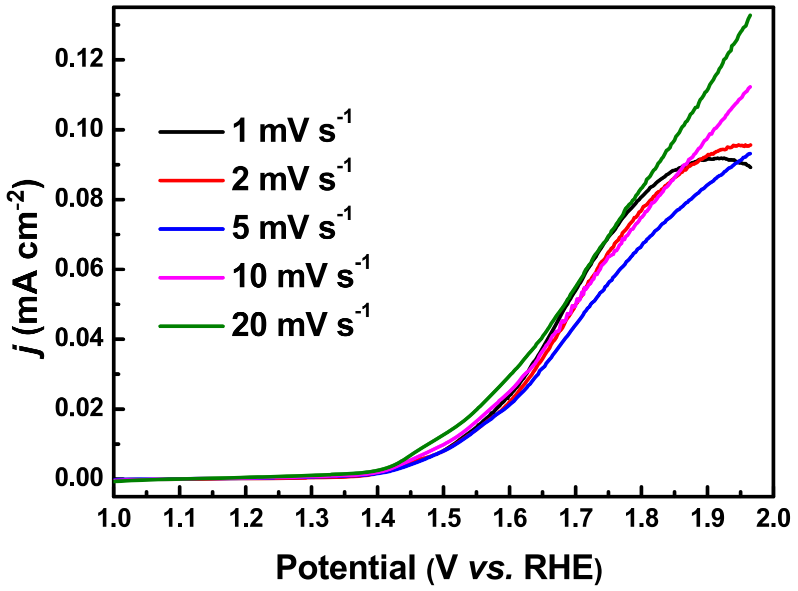

Figure S5. LSV profile of Cu_2WS_4 at various scan rates in 0.1 N KOH.

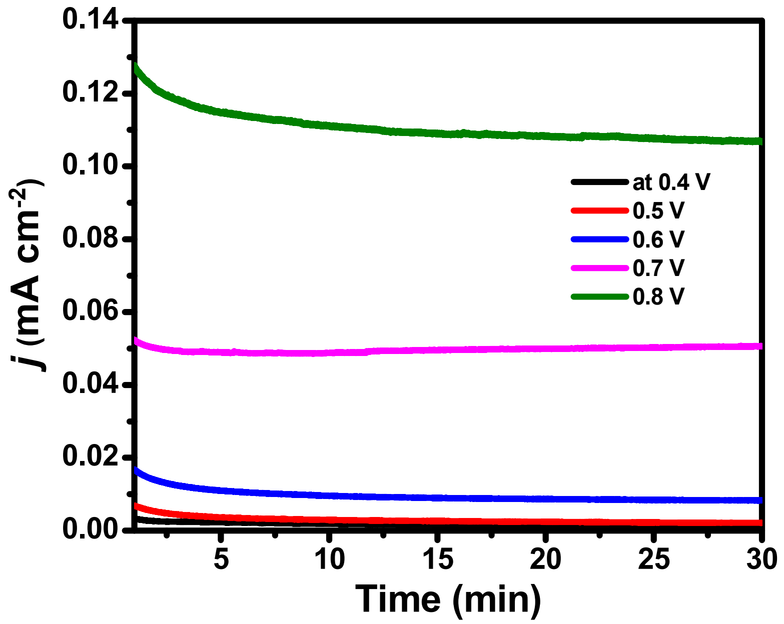

Figure S6. Potentiostatic chronoamperometric study of Cu_2WS_4 at various potential (V vs Ag/AgCl).

The open-source software Quantum ESPRESSO (QE) was used to undertake first-principles calculations of the density of states (DOS) for Cu_2WS_4 [1,2]. A plane-wave kinetic energy cut-off of 950 eV was chosen. Calculations were performed on the 14 atoms in the cell with tetragonal symmetry. Monkhorst-Pack unique k-point grids with dimensions of 12x12x6 were used to sample the Brillouin zone. the calculated total and partial density of state of Cu_2WS_4 are shown in Figure 5. It can be seen that the valence band (VB) near the Fermi level was mostly composed of Cu 3d and S 3p orbitals, whereas S 3p and W 5d orbitals dominantly constituted the conduction band. Hence, it could be concluded that Cu 3d and W 5d orbital might be contributing in OER electrocatalysis whilst S 3p orbitals are primarily responsible for the HER electrocatalysis.

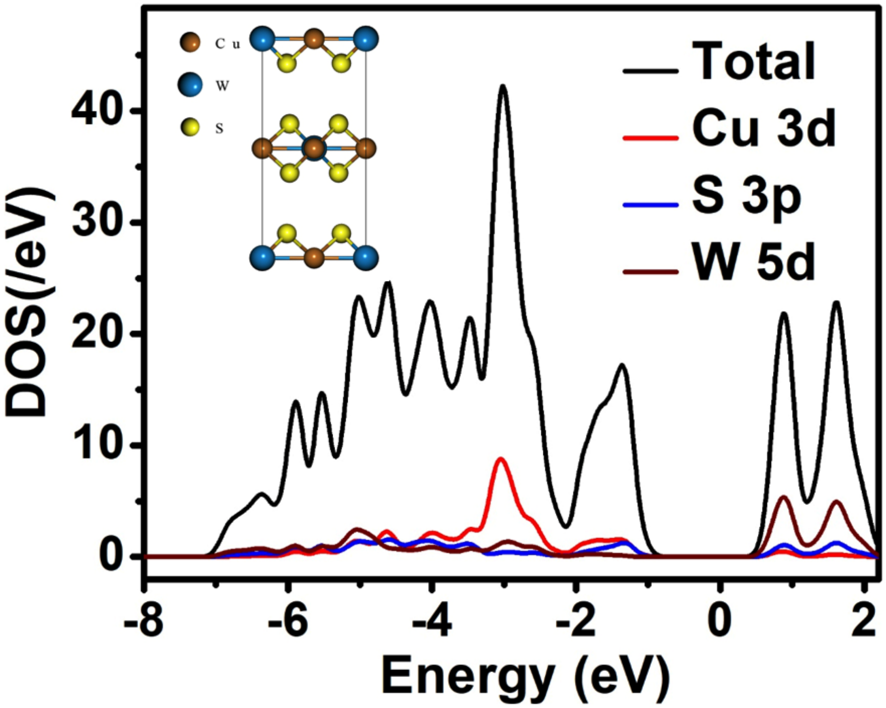

Figure S7. Density of states (DOS) and partial DOS plots for Cu_2WS_4 (inset: Showing the unit cell structure for which DOS was calculated).

Spectrum processing :

Peaks possibly omitted : 0.263, 0.520 keV

Processing option : All elements analyzed (Normalised)

Number of iterations = 3

Standard :

S FeS2 1-Jun-1999 12:00 AM

Cu Cu 1-Jun-1999 12:00 AM

W W 1-Jun-1999 12:00 AM

Element	Weight%	Atomic%
S K	30.88	61.43
Cu K	22.21	22.29
W M	46.91	16.27
Totals	100.00	

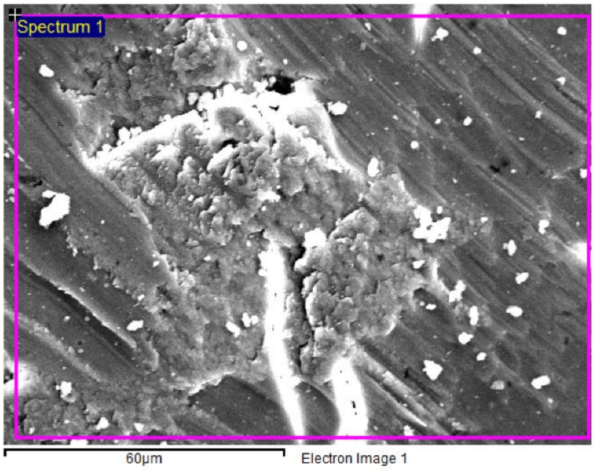

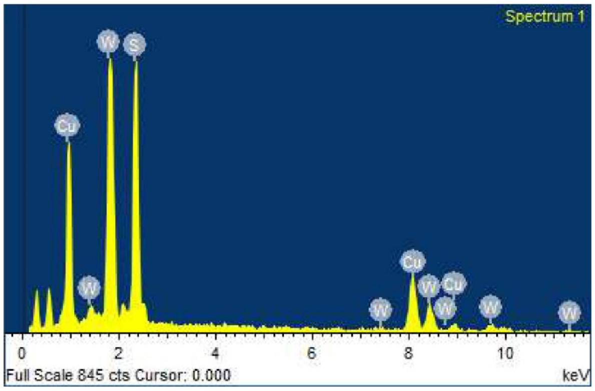

Comment:

Figure S8. EDAX analysis for Cu_2WS_4.

References

[1] P Giannozzi, O Andreussi, T Brumme, O Bunau, M Buongiorno Nardelli, M Calandra, R Car, C Cavazzoni, D Ceresoli, M Cococcioni, N Colonna, I Carnimeo, A Dal Corso, S de Gironcoli, P Delugas, R A DiStasio Jr, A Ferretti, A Floris, G Fratesi, G Fugallo, R Gebauer, U Gerstmann, F Giustino, T Gorni, J Jia, M Kawamura, H-Y Ko, A Kokalj, E Küçükbenli, M Lazzeri, M Marsili, N Marzari, F Mauri, N L Nguyen, H-V Nguyen, A Otero-de-la-Roza, L Paulatto, S Poncé, D Rocca, R Sabatini, B Santra, M Schlipf, A P Seitsonen, A Smogunov, I Timrov, T Thonhauser, P Umari, N Vast, X Wu and S Baroni, J.Phys.:Condens.Matter 29, 465901 (2017)

[2] P. Giannozzi, S. Baroni, N. Bonini, M. Calandra, R. Car, C. Cavazzoni, D. Ceresoli, G. L. Chiarotti, M. Cococcioni, I. Dabo, A. Dal Corso, S. Fabris, G. Fratesi, S. de Gironcoli, R. Gebauer, U. Gerstmann, C. Gougoussis, A. Kokalj, M. Lazzeri, L. Martin-Samos, N. Marzari, F. Mauri, R. Mazzarello, S. Paolini, A. Pasquarello, L. Paulatto, C. Sbraccia, S. Scandolo, G. Sclauzero, A. P. Seitsonen, A. Smogunov, P. Umari, R. M. Wentzcovitch, J. Phys. Condens. Matter **21**, 395502 (2009). URL http://www.quantum-espresso.org".
